# FTH1P3, a Novel H-Ferritin Pseudogene Transcriptionally Active, Is Ubiquitously Expressed and Regulated during Cell Differentiation

**DOI:** 10.1371/journal.pone.0151359

**Published:** 2016-03-16

**Authors:** Maddalena Di Sanzo, Ilenia Aversa, Gianluca Santamaria, Monica Gagliardi, Mariafranca Panebianco, Flavia Biamonte, Fabiana Zolea, Maria Concetta Faniello, Giovanni Cuda, Francesco Costanzo

**Affiliations:** 1 Research Center of Advanced Biochemistry and Molecular Biology, Department of Experimental and Clinical Medicine, Magna Graecia University of Catanzaro, Salvatore Venuta Campus, Catanzaro, Italy; 2 IBFM-CNR, c/o Università Magna Graecia, Catanzaro, Italy; North Carolina State University, UNITED STATES

## Abstract

Ferritin, the major iron storage protein, performs its essential functions in the cytoplasm, nucleus and mitochondria. The variable assembly of 24 subunits of the Heavy (H) and Light (L) type composes the cytoplasmic molecule. In humans, two distinct genes code these subunits, both belonging to complex multigene families. Until now, one H gene has been identified with the coding sequence interrupted by three introns and more than 20 intronless copies widely dispersed on different chromosomes. Two of the intronless genes are actively transcribed in a tissue-specific manner. Herein, we report that FTH1P3, another intronless pseudogene, is transcribed. FTH1P3 transcript was detected in several cell lines and tissues, suggesting that its transcription is ubiquitary, as it happens for the parental ferritin H gene. Moreover, FTH1P3 expression is positively regulated during the cell differentiation process.

## Introduction

It has been estimated that the human genome contains more than 15,000 pseudogenes [1], and that the majority of them, called processed pseudogenes, derive from the retrotranscription of the mature mRNA [2]. After being considered for many years devoid of functions, so as to be identified as “junk DNA”, the role of pseudogenes in controlling gene expression has been recently revealed in normal and transformed cells [3, 4]. Some pseudogenes are actively transcribed but not translated because the RNA harbours premature stop codons and/or other mutations that abrogate the synthesis of the functional proteins [5]. A growing body of evidence indicates that these non-coding RNAs (ncRNAs) regulate, at post-transcriptional level, the expression of their cognate protein-coding genes acting as antisense RNAs or endogenous small-interference RNAs (endo-siRNA or esiRNA), or as decoy for miRNA and for RNA-binding proteins [6, 7]. The transcript of the pseudogene TUSC2P, for example, regulates the expression of the cognate TUSC2 mRNA by interacting with endogenous miRNAs [8]. The same mechanism is active in the regulation of HMGA1 oncogene [9].

Ferritin plays a pivotal role in the intracellular iron metabolism, due to its ability in sequestering free iron in a non-toxic and bio-available form [10]. In eukaryotes the protein, of 450 kDa, is localized in cytoplasm, nucleus and mitochondria [10, 11].

Two distinct genes, subjected to different transcriptional control mechanisms, code the light and heavy chains of cytoplasmic ferritin [12–14]. The gene for the ferritin light chain (FTL) is located on chromosome 19 [15], while the ferritin heavy chain (FHC, FTH) gene maps on chromosome 11 [16], with the coding sequence interrupted by three introns, whose position is highly conserved among the different species [17]; with the sole exception of the chicken, multiple copies of FHC gene are present in these genomes [13, 18, 19]. The majority of the FHC loci, widely dispersed within the genome, shows similar structural features; the absence of introns, the presence of direct repeats at the 5’ and 3’ends as well as the remnant of a polyA tail allowed their identification as retro-transcribed pseudogenes [13, 20, 21]. Two of the FHC intronless copies are transcribed and give rise to functional peptides, namely FTHL17 (Ferritin heavy polypeptide-like 17) [22] and the one coding for mitochondrial ferritin (FtMt) [11]. Both appear to be transcribed exclusively or preferentially in restricted cell types. Very recently, it has been demonstrated that FTH1P3 (Ferritin heavy polypeptide 1 pseudogene 3), another member of the FHC gene family, is transcribed in oral squamous cell carcinoma [23].

In this work we evaluated FTH1P3 expression levels in different human cell lines and tissues. The results indicate that the pseudogene is ubiquitously expressed and that the transcript is positively modulated during cell differentiation. The steady-state amounts of FTH1P3 and of its parental FHC gene does not appear to be reciprocally related.

## Materials and Methods

### Cell culture and tissue samples

The human cell lines SKOV-3 (ATCC number HTB-77), K562 (ATCC number CCL-243), HCC1937 (ATCC number CRL-2336), H460 (ATCC number HTB-177) were cultured in RPMI 1640 medium supplemented with 10% fetal bovine serum, 10 units/ml penicillin and 10 mg/ml streptomycin at 37°C in a humidified 5% CO_2_ atmosphere. The human cell lines Capan (ATCC number HTB-79), HeLa (ATCC number CCL-2), MCF-7 (HTB-22), Caco-2 (ATCC number HTB-37) were cultured in DMEM medium supplemented with 10% fetal bovine serum, 10 units/ml penicillin and 10 mg/ml streptomycin at 37°C in a humidified 5% CO_2_ atmosphere. The LxF-289 cells (DSMZ number ACC-265) were cultured in HAM’S F10 medium supplemented with 10% fetal bovine serum, 10 units/ml penicillin and 10 mg/ml streptomycin at 37°C in a humidified 5% CO_2_ atmosphere. Okadaic acid was added directly to the MCF-7 culture medium at a final concentration of 100 nM for 4 hours. Hemin was added to the K562 culture medium at a final concentration of 50 μM as described [24] for 72 h.

Tissue samples were obtained from six affected patients after obtaining written consent.

### Preparation of lentiviral supernatants and transduction of MCF-7 cells and H460 cells

Lentiviral preparations and transductions were performed as previously described [24, 25]. Briefly, 5x10^6^ HEK-293T cells were grown on 10-cm plates to 70–80% confluence and co-transfected with 10 μg shRNA lentiviral DNA, 2 μg pCMV-VSV-G expressing envelope plasmid, and 18 μg packaging viral CMV Δ8.9 plasmid, using the calcium phosphate precipitation method. Eight hours later, fresh medium was added and cells were cultured for an additional 2 days. The medium was harvested 48h post-transfection and filtered through a 0.45 μm filter. The supernatant from HEK-293T cultures were used to cross-transduce MCF-7 and H460 cells in the presence of 8 μg/ml polybrene (Sigma) and positive clones were isolated by puromycin selection (1 μg/ml). Cells were stably transduced with a lentiviral DNA containing either an shRNA that targets the 196–210 region of the FHC mRNA (sh29432) (MCF-7 shFHC, H460 shFHC), or a control shRNA without significant homology to known human mRNAs (MCF-7 shRNA, H460 shRNA).

### RNA extraction, reverse-transcription PCR and quantitative real-time PCR

Total RNA was isolated by TRIzol (Invitrogen Life Technologies) and treated with 2 Units/μl of Turbo DNase for 30 minutes at 37°C. Subsequently, cDNA synthesis was performed with a reverse transcription kit (Invitrogen) [26]. PCR analysis of GAPDH mRNA was performed as previously described [27], utilising 50 ng of cDNA as template. Two hundreds and fifty ng of cDNA were utilised for U6 transcript amplification with the following primers: forward 5′-gtg ctc gcttcg gca gca cat ata c-3′; reverse 5′-aaa aat atg gaa cgc ttc acg aat ttg-3′. Two hundreds and fifty ng of cDNA were utilised for FTH1P3 transcript amplification with the following primers: ΨF 5′-ctg tgc ggg gag aat gc-3′; ΨR 5′-cca aat gta atg caa aat g-3’. Two additional primers (HF and HR) were designed to be used in combination with ΨR and ΨF, respectively. HF 5’-cct cca ttt acc tgt gcg tg-3’; HR 5’-tgg ggg tca ttt ttg tca-3’. PCR conditions are shown in Table 1.

**Table 1:** PCR conditions used in this work.

PRIMERS	Step 1	Step 2 (35 cycles)			Step 3
	Enzyme activation Time:2 min	Denaturation Time: 50 sec	Annealing Time: 50 sec	Extension Time: 50 sec	Final extension Time:10 min
ΨF/ΨR	94°C	94°C	43°C	72°C	72°C
HF/ΨR	94°C	94°C	43°C	72°C	72°C
ΨF/HR	94°C	94°C	57°C	72°C	72°C
U6F/U6R	94°C	94°C	48°C	72°C	72°C

The amplified products were electrophoresed on 1.5% agarose gel for GAPDH mRNA or 3% agarose gel for FTH1P3 and U6 transcripts.

qPCR of FHC, FTH1P3, γ-globin, GAPDH and U6 were performed with the above described primers in a final volume of 20 μl containing cDNA, 1x Power SYBR Green PCR Master mix (Life Technologies), 20 pmol of each primer pair and nuclease-free water. The primers for γ-globin were: forward 5′-cag aaa tac aca tac aca ctt cc-3′; reverse 5′-gag aga tca cac atg att ttc tt-3′. The cycling conditions were: 95°C for 10 min followed by 40 cycles at 95°C for 10s, 60°C for 10s for FHC, γ-globin and GAPDH, 43° for 10s for FTH1P3 and U6, 72°C for 20s. Data analysis was performed using the 2^-ΔΔCt^ method [28].

### DNA sequence analysis

The 87-bp, 149-bp, 260-bp cDNA fragments corresponding to FTH1P3 and the 117-bp cDNA fragment corresponding to FHC were run on a 3% agarose gel and purified using the gel purification kit (Qiagen). Direct sequence analysis was performed in both directions using ABI PRISM Big-Dye Terminator Kit (Applied Biosystems), and run on the ABI-3100 genetic Analyzer (Applied Biosystems). The DNA sequence was further analysed by the nucleotide BLAST program and the BioEdit software.

### Total protein extraction and quantification of Alkaline Phosphatase activity (ALP) in Caco-2 cells

Caco-2 cells were harvested at 3, 7 and 14 days and lysed in a proper amount of RIPA buffer (50 mM Tris-HCl pH 7.4, 1% NP-40, 0.5% Na-deoxycholate, 0.1% SDS, 150 mM NaCl, 2 mM EDTA, 50 mM NaF) containing 1 mM PMSF and complete protease inhibitor mixture. The total protein concentration of individual lysate was determined by Bradford protein assay kit (Biorad). ALP activity was analyzed by using an enzymatic assay (Cobas 6000 Roche, Switzerland).

### In silico analysis of gene-miRNA interaction

The analysis of miRNAs potentially targeting the 3’UTRs of FHC and FTH1P3 was performed by using the miRWalk 2.0-database software [29].

### Statistical analysis

Data analysis was done by Student’s *t*- test assuming equal variances. Data are the mean (±SD) of three independent experiments.

## Results and Discussion

A survey of the literature, integrated with the data present on NCBI Ref-Seq database, reveals that the human genome contains, dispersed on different chromosomes, more than 20 FHC gene copies. The presence of typical structural features, such as a polyA tail and the absence of intronic sequences allows the allocation of most of the FHC gene copies to the category of the so-called processed or retro-transposed pseudogenes [13, 20, 21]. Along with one gene, provided of three introns and ubiquitously expressed [13, 30], two of the intronless loci are transcribed and translated, namely FTHL17, coding for a low stability H-chain protein without ferroxidase activity [22], and the one coding for mitochondrial ferritin (FtMt) [11]. In this work, we focused our attention on FTH1P3, located on chromosome 2, since it is the only one indicated on NCBI Ref-Seq database as “provisional RNA sequence”, thus suggesting that it might be transcribed. Indeed, in a very recent publication, FTH1P3 has been identified in a group of 41 up-regulated long non-coding RNAs (lncRNAs) in oral squamous cell carcinoma [23]. However, to our knowledge, no other reports on its expression levels and cell/tissue specificity are available. Consequently, we decided to evaluate the presence of the FTH1P3 transcript in different human cell lines and tissues by means of reverse-transcription PCR and qPCR.

### Setting of the conditions for FTH1P3 selective amplification

**Fig 1:**
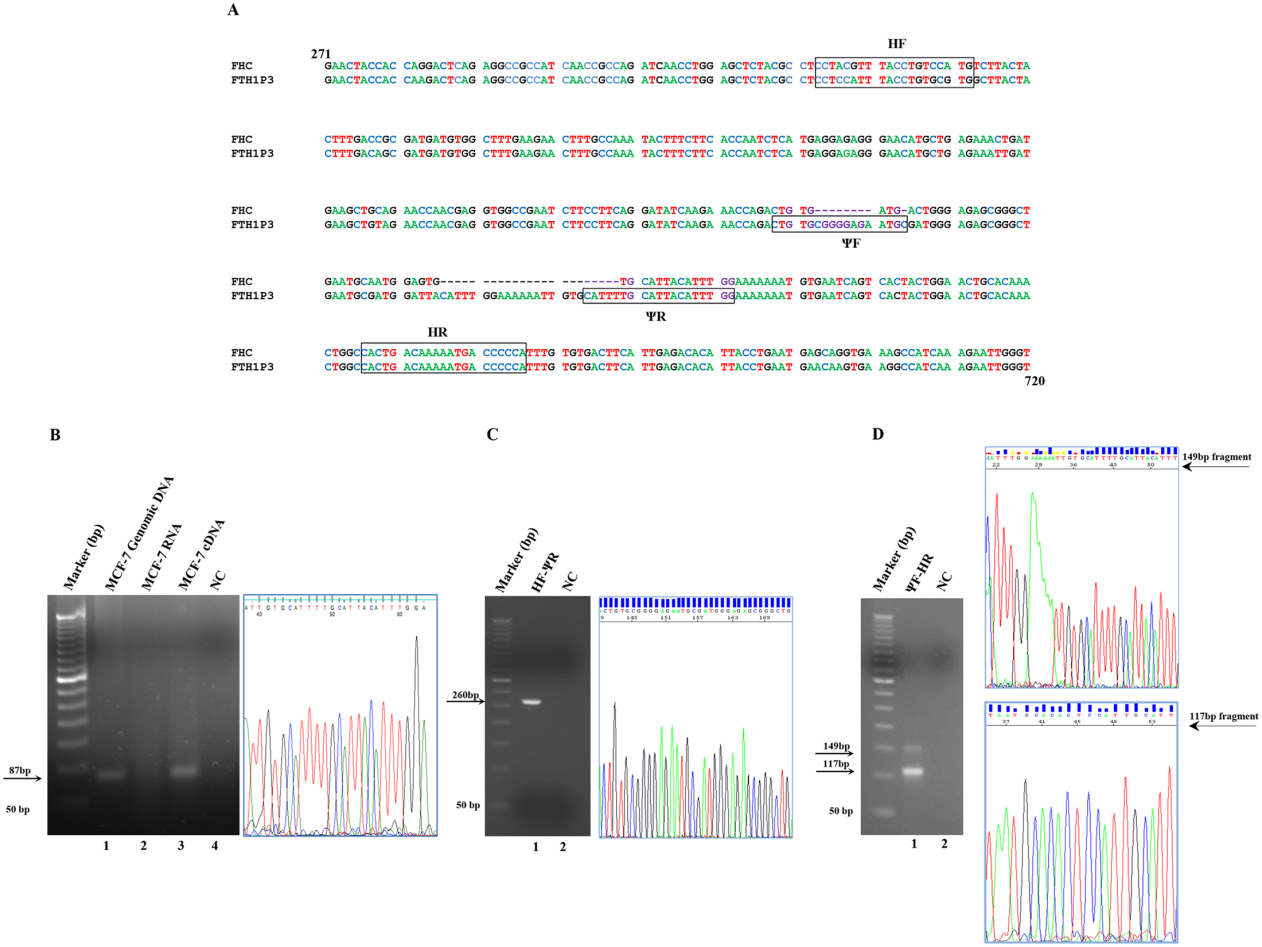
Panel A. Alignment of the sequences of the FHC mRNA and the FTH1P3 pseudogene from nt. 271 to nt. 720. The FHC accession number is NM_002032.2. The FTH1P3 accession number is NR_002201.1. The ΨF, ΨR, HF and HR oligonucleotide sequences are boxed. Panel B. PCR analysis of FTH1P3 transcript, performed with ΨF and ΨR oligonucleotides. Lane 1: genomic DNA from MCF-7 cells; lane 2: DNaseI-treated total RNA from MCF-7 cells; lane 3: cDNA from MCF-7 cells; lane 4: negative control without template (NC). The predicted PCR product, indicated by an arrow, is 87-bp long. DNA sequencing electropherogram (nt. 38 to nt. 63) of the FTH1P3 fragment. Panel C. PCR analysis of FTH1P3 transcript, performed with HF/ΨR (lane 1), negative control without template (lane 2). The predicted PCR product, indicated by arrow is 260-bp long. DNA sequencing electropherogram (nt. 139 to nt. 173) of the 260-bp FTH1P3 fragment. Panel D. PCR analysis of FTH1P3 transcript elongated with ΨF/HR oligonucleotides (lane 1), negative control without template (lane 2). The predicted PCR products, indicated by arrows, are 149-bp for FTH1P3 and 117-bp for FHC. DNA sequencing electropherogram (nt. 20 to nt. 54) of the 149-bp FTH1P3 fragment and of the 117-bp FHC fragment (nt. 35 to nt. 55).

One of the major glitches in analysing pseudogene expression is represented by the high degree of sequence homology among the expressed gene and its copies, as well as among the different pseudogenes. This issue might be particularly significant in the case of the ferritin multigene family, composed by many highly homologous members [17]. The full alignment of FTH1P3 with FHC mRNA, provided as S1 Fig, demonstrates that the two sequences share a 92% identity; however, FTH1P3 presents, in position 513 and 556, two extra-sequences 8 and 23 nucleotides long, respectively. These two extra-sequences, most likely derived from internal duplication and insertional events, are also missing in all the other pseudogenes; this finding allowed the drawing of a pair of PCR primers, identified as ΨF and ΨR in Panel A of Fig 1, highly specific for FTH1P3. To test the primers, we performed a reverse-transcription PCR on the total RNA extracted from the human breast adenocarcinoma cell line MCF-7 after an extensive DNaseI treatment (compare the PCR amplification of the DNaseI-treated RNA in lane 2 with that of cDNA in lane 3 of Fig 1, Panel B). The oligonucleotides directed the synthesis of a fragment of the expected size (87-bp) that has been extracted from the agarose gel and subjected to sequencing to confirm its uniqueness (Panel B of Fig 1). To further demonstrate that the full-length pseudogene is actually transcribed in MCF-7 cells, we also designed a pair of primers covering a longer section of the transcript, indicated as HF and HR in Panel A of Fig 1. The HF and HR primers are positioned, respectively, 155 bp upstream and 43 bp downstream the 87-bp FTH1P3 region previously described. The HF oligonucleotide, utilised in combination with ΨR, gives rise to an amplicon of the expected size, i. e. 260-bp, shown in Panel C of Fig 1, together with its nucleotide sequence. In the conditions we set up (see Materials and Methods), the ΨF/HR pair of oligonucleotides directed the synthesis of two fragments of 149-bp and 117-bp corresponding, respectively, to the FTH1P3 transcript and to the FHC mRNA. The amplification of the two fragments, along with their DNA sequence, is shown in Panel D of Fig 1. The complete DNA sequences of the 260-bp, of the 149-bp and of the 117-bp are provided in S2, S3 and S4 Figs, respectively. Finally, it must be emphasized that, while 50 ng of input cDNA are sufficient to amplify the FHC transcript, at least 250 ng of cDNA are needed to uncover the presence of the FTH1P3 transcript, suggesting that expression levels of FHC and FTH1P3 mRNAs are consistently different (compare also the relative intensity of the pseudogene and FHC amplicons in Panel D of Fig 1).

### FTH1P3 is transcribed in different human cell lines

**Fig 2:**
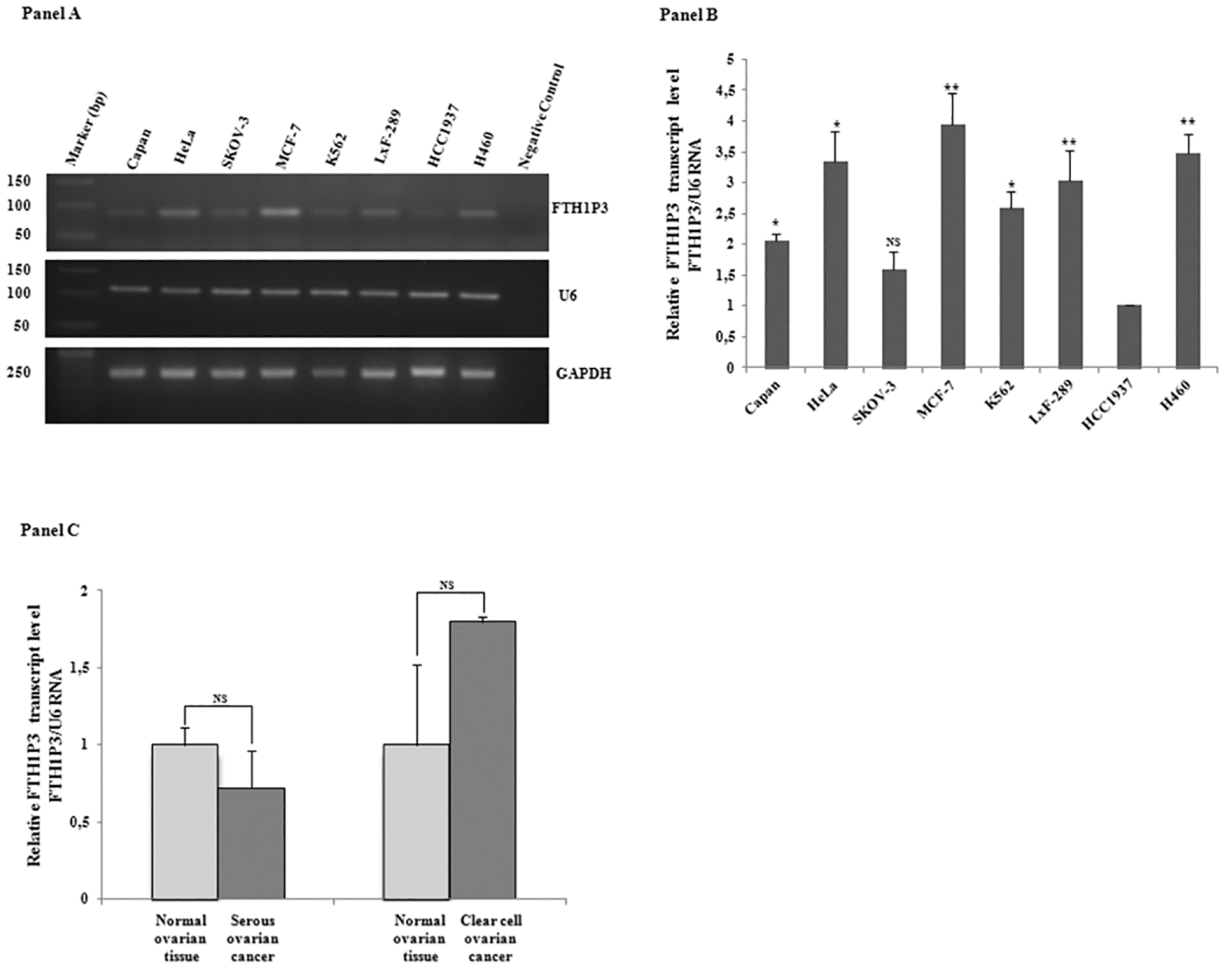
Panel A. RT-PCR analysis of FTH1P3 transcript in the cancer cell lines Capan, HeLa, SKOV-3, MCF-7, K562, LxF-289, HCC1937, H460. U6 and GAPDH transcripts were used as reference genes. Panel B. qPCR analysis of FTH1P3 in the cancer cell lines; U6 RNA is the reference gene. Data are the mean ± SD of three independent experiments (*p<0.05; **p<0.001). Panel C. qPCR analysis of FTH1P3 in serous and clear cell ovarian cancer and in normal tissue counterparts. Data are the mean ± SD of three independent experiments. NS: p value not significant.

Afterwards, by using the ΨF and ΨR oligonucleotide pairs, we analysed FTH1P3 expression by reverse-transcription PCR on RNAs obtained from 8 different human cell lines derived from different tissues (Capan, HeLa, Skov3, MCF-7, HCC1937, LxF-289 and H460) as well as from the haematological compartment (K562). Panel A of Fig 2 shows the agarose gel that confirms the presence of the expected 87-bp product in the samples and demonstrates that FTH1P3 transcript is indeed expressed in all the cell lines. U6 and GAPDH, used as reference genes, show a uniform degree of expression among all the samples. In the subsequent experiments we therefore utilised U6 as reference gene for FTH1P3, taking advantage of the similar qPCR conditions, while GAPDH was the reference gene for FHC mRNA.

Next, a qPCR assay was performed to define FTH1P3 relative abundance. Pseudogene amounts were quantified by using 2^-ΔΔCt^ method, and normalised to those of the low FTH1P3-expressing HCC1937 cells. The quantitative analysis, shown in Panel B of Fig 2, reveals differences of the order of 4-fold among these cells, with the highest value detected in MCF-7, followed by HeLa and H460. FTH1P3 was also detected in a serous ovarian cancer sample and in the corresponding normal tissue, as well as in a clear cell ovarian cancer and its normal counterpart (Panel C of Fig 2). In contrast to the findings reported by Zhang et al. on oral squamous cell carcinoma [23], ovarian cancer does not display significant differences in the relative abundance of the pseudogene transcript in comparison with normal tissues.

The detection of FTH1P3 transcript in all cells and tissues analysed strongly suggests that this pseudogene might be ubiquitously expressed, unlike the vast majority of the other pseudogenes, which are frequently transcribed in a tissue-specific manner [3, 4]. It is noteworthy that FTHL17 is expressed only in testis [22], just as it is restricted to a few tissues the transcription of the intronless locus coding for FtMt [11].

### The amounts of FTH1P3 and FHC transcripts are not reciprocally related

**Fig 3:**
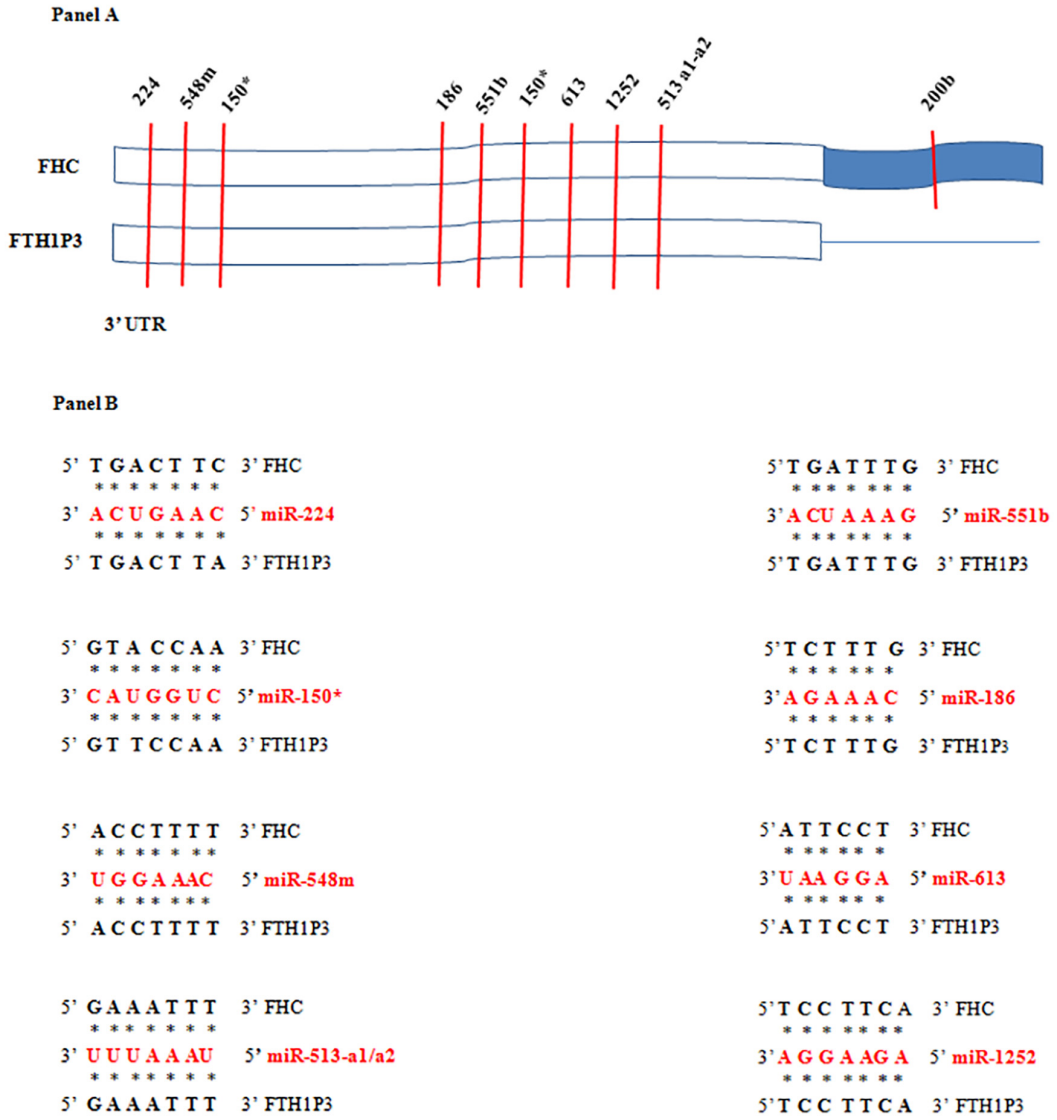
MRE *in silico* analysis. Panel A. The 3’UTRs of FHC and FTH1P3 contain a highly conserved domain (in white) in which several common MRE, indicated by vertical bars, have been identified. The corresponding miRNAs are indicated above the vertical bars. Panel B. Binding of common targeting miRNAs to FHC and FTH1P3 transcripts.

As already reported, FTH1P3 has been recently classified as lncRNA [23], most probably because of the presence of multiple early stop codons downstream the AUG that would prevent translation of a functional peptide. The FTH1P3 *in silico* translation, showing the presence of several early stop codons, the first of which located 220 nucleotides downstream the AUG, is provided as S5 Fig. In the most recent years, several studies have revealed that the lncRNAs, and among them the expressed pseudogenes, might control the expression of both parental and non parental genes in the context of the competing endogenous RNA (ceRNA) networks [6, 31]. When transcribed from the same DNA strand, the pseudogene transcript might act as molecular trap for miRNAs and/or for RNA-binding proteins [32]. The prerequisite for this regulation is the coexistence, in the same cell, of both the gene and pseudogene transcripts. We reasoned that: i) FTH1P3 and FHC are both encoded by the same strand on chromosome 2 and 11, respectively; ii) FTH1P3 is expressed in many different cell types, as it happens for FHC; iii) FHC expression is regulated, among other mechanisms, by a miRNA Response Element (MRE) in the 3’UTR, recognised by miR-200b [33]. Since the 3’UTR of FTH1P3 is shorter than that of FHC (see S1 Fig) and does not contain the seed region for miR-200b, we performed *in silico* analysis for MRE identification in the 3’UTR common to the two transcripts. Within this region, which shares a 92% of homology, we identified conserved seed matches for 8 miRNAs, including 150*, 548m, 613 and 1252, perfectly conserved in both the transcripts (Panels A and B of Fig 3).

**Fig 4:**
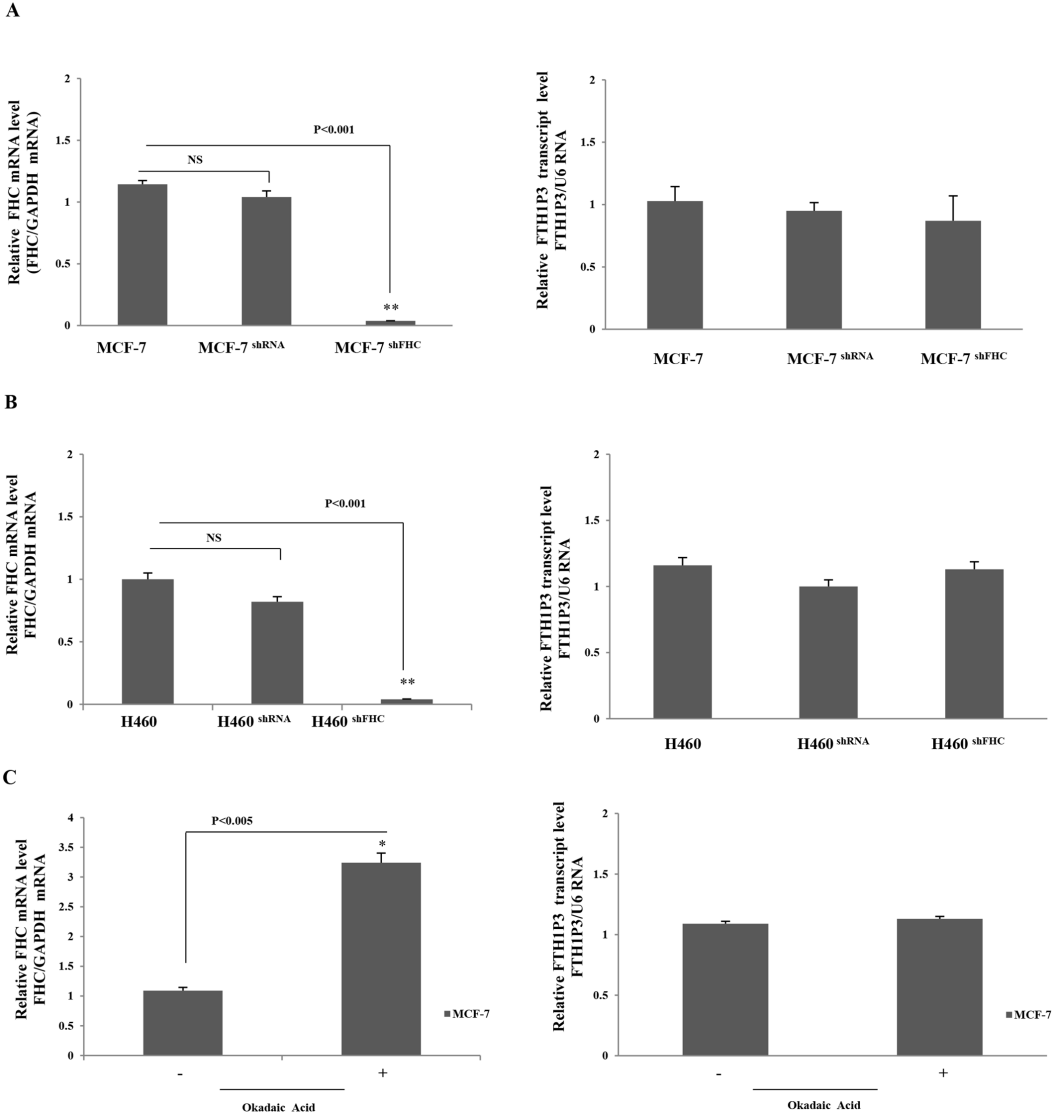
Panel A. qPCR of FHC and FTH1P3 transcripts in wt, shRNA and shFHC transduced MCF-7 cells. GAPDH was used as internal control for FHC and U6 for FTH1P3. Panel B. qPCR of FHC and FTH1P3 transcripts in wt, shRNA and shFHC transduced H460 cells. GAPDH was used as internal control for FHC and U6 for FTH1P3. Panel C: qPCR of FHC and FTH1P3 in MCF-7 cells treated and untreated with okadaic acid. Data are the mean ± SD of three independent experiments (*p<0.005; **p<0.0001). NS: p value not significant.

Taken together, these considerations prompted us to investigate the existence of a cross-talk between FHC and FTH1P3 transcript levels by modulating the endogenous FHC expression in two cell lines of epithelial origin, namely MCF-7 and H460 cells, where FTH1P3 transcript is expressed at levels higher than in the other cells. In order to knock-down FHC expression, we utilised an shRNA (sh29432), already described [24, 25, 34] that perfectly targets the 196**–**210 region of the FHC mRNA. This shRNA harbours three critical mismatches with the sequence of FTH1P3, therefore preventing the annealing with the pseudogene transcript. The steady-state amounts of FTH1P3 were evaluated in a pool of stable MCF-7 and H460 transfectants, and compared to those found in the wild type cells and in cells transfected with a scrambled shRNA. The results of a triplicate set of independent experiments indicate that the knockdown of the parental FHC gene does not significantly modify the FTH1P3 amounts in both the MCF-7 (Fig 4 Panel A), and H460 (Fig 4 Panel B) cell systems.

We also induced an up-regulation of the endogenous FHC mRNA by treating MCF-7 cells with Okadaic Acid (OA), a tumor-promoting agent able to activate the FHC transcription [35]. The results indicate that 4 hours OA treatment was able to induce a significant increase of FHC mRNA in MCF-7 cells, leaving unchanged the FTH1P3 levels (Fig 4, Panel C). Expression levels of the U6 and GAPDH reference genes were unaltered in all these experimental conditions (p values not statistically significant).

These results suggest that interference between FHC and FTH1P3 transcripts is unlikely to occur, at least with respect to their reciprocal stability and consequently their relative abundance. As suggested by our experiments, FTH1P3 might be considered a low-copy number transcript, while the FHC mRNA is abundantly expressed in normal and transformed cells [30]. We retain that the low FTH1P3/FHC transcript ratio might prevent transcripts interference, as it has been described for CYP19P1 [36].

This finding, however, does not exclude the possibility that additional post-transcriptional mechanisms may engage FTH1P3 and FHC transcripts. Ferritin, in fact, is strongly regulated at the translational level by the binding of RNA-binding proteins (IRE-bp1 and IRE-bp2) to a stem-loop structure located in the 5’ UTR of the mRNA [37]. We noticed that also FTH1P3 harbours, in its 5’ UTR, a perfectly conserved stem-loop IRE sequence (underlined in S1 Fig), potentially able to be recognised by IRE-bp. Thus, FTH1P3 transcript might compete, in specific cell conditions, with FHC mRNA for IRE-bp binding, therefore enhancing the translational efficiency of FHC. This mechanism might be specific for FTH1P3. Even though an almost perfectly conserved IRE sequence is present in the majority of the untranscribed FHC pseudogenes, FTHL17, which is transcribed and translated and possibly plays regulatory functions (22) harbours an IRE sequence only preserved to 40%. Further analyses are needed to address this point.

### FTH1P3 is positively regulated during cell differentiation

**Fig 5:**
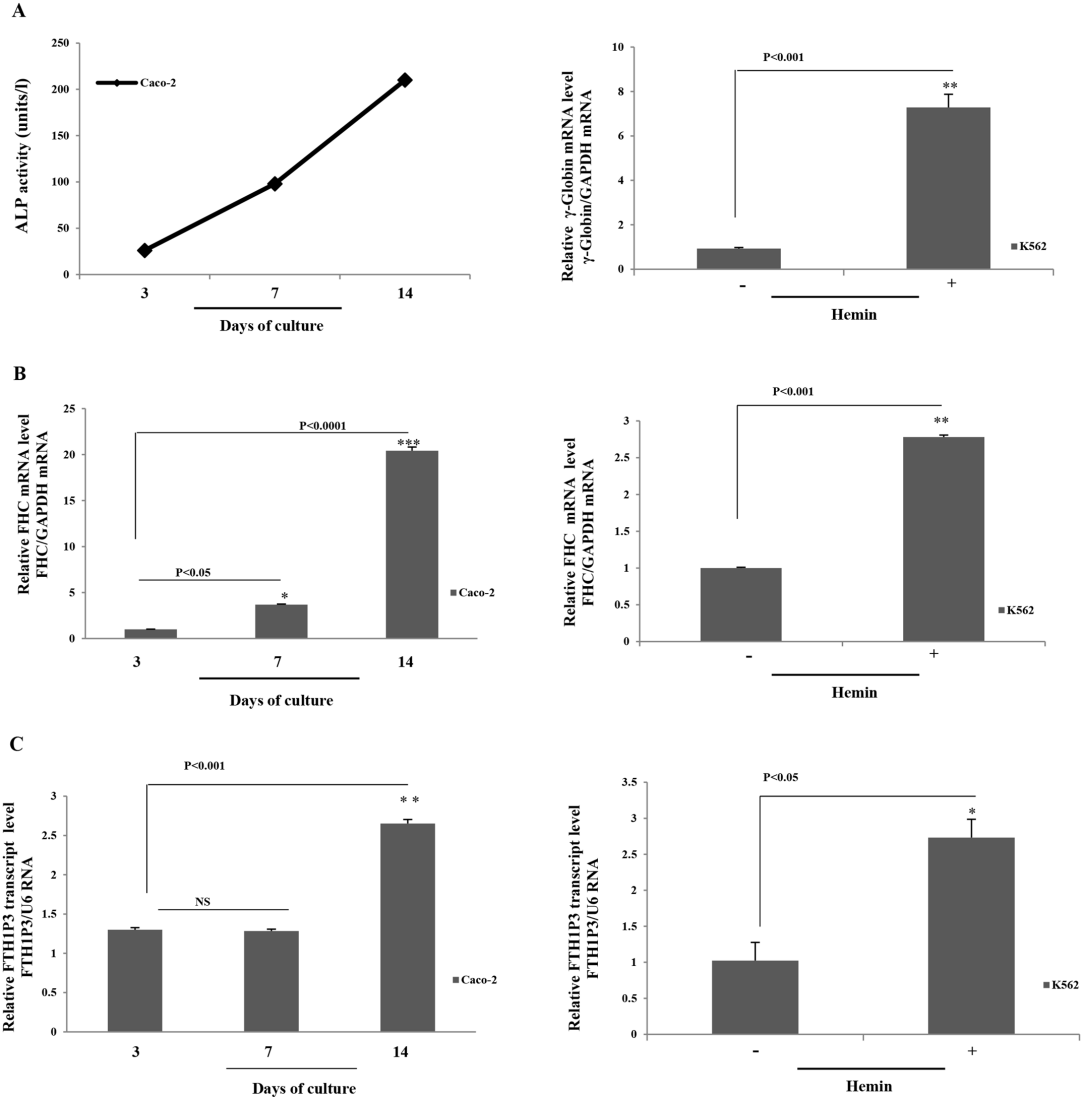
Panel A. ALP activity in Caco-2 cells and qPCR analysis of γ-globin mRNA in K562 cells. Panel B. qPCR analysis of FHC mRNA in Caco-2 and in K562 cells. Panel C. qPCR analysis of FTH1P3 transcript in Caco-2 and in K562 cells. Data are the mean ± SD of three independent experiments (*p<0.05; **p<0.001; ***p<0.0001). NS: p value not significant.

Expressed pseudogenes might be important regulators in the processes of cellular differentiation and neoplastic transformation [38]. As an example of their key role, it can be mentioned the regulatory relationship between the transcription factor OCT4 and its expressed pseudogenes during the differentiation of human embryonic stem cells (ESCs); moreover, only the OCT4 pseudogenes appear to be expressed in a variety of differentiated cells [39]. Notably, the other ferritin intronless gene, FTHL17, is down regulated during the differentiation of mouse ESC in monolayers [22]. We thus decided to investigate whether FTH1P3 expression is modulated during cell differentiation. As a model system, we choose the human colon carcinoma cell line Caco-2 and the human erythroleukemia cells K562. Caco-2 cells spontaneously differentiate into enterocyte-like cells depending exclusively on cell density [40], while K562 cells erythroid differentiation can be triggered by hemin [24]. We have previously demonstrated that the differentiation process is paralleled by an accumulation of the FHC mRNA in both cell lines [24, 41]. The results are shown in Fig 5. As differentiation markers we evaluated the alkaline phosphatase activity in the case of Caco-2 cells [42] and the γ-globin amounts for K562 cells [24] (Fig 5 Panel A). In panel B is reported the qPCR of FHC mRNA, confirming that its steady-state amounts are increased in the fully differentiated cells. In Panel C is reported the qPCR of FTH1P3 transcript: as well as the FHC mRNA, also that of the pseudogene accumulates when the differentiation process is fully completed in both the cell lines, with an increase of about three-fold in comparison with the undifferentiated cells. Again, we verified that the expression levels of the U6 and GAPDH reference genes were unchanged through differentiation (p values not statistically significant).

According to the results previously reported, showing the absence of a cross-talk between FHC and FTH1P3 transcripts, we believe that the increased levels of the pseudogene RNA during differentiation are independent from those of ferritin, but might be rather linked to specific functions, yet to be explored.

## Conclusions

Taken all together, the data presented in this work indicate that FTH1P3, an intronless member of the FHC gene family, is expressed along with the ones coding for FtMt and FTHL17. Compared to them, FTH1P3 shows two substantial differences: FTHL17 and FtMt loci are subjected to a tightly cell-specific transcription and are translated into a functional protein, while FTH1P3 is widely expressed in a variety of cells, as it happens for the parental FHC gene, and most likely does not code for a peptide product. On the other hand, the ubiquitous expression of FTH1P3, as well as its positive regulation during the process of cell differentiation, strongly suggests that this pseudogene may play a role in the complex regulatory network of the ferritin gene family.

## Supporting Information

S1 Fig Full alignment of FHC mRNA and FTH1P3 transcripts. (TIF)

S2 Fig DNA sequencing electropherogram of 260-bp fragment. (TIF)

S3 Fig DNA sequencing electropherogram of 149-bp fragment. (TIF)

S4 Fig DNA sequencing electropherogram of 117-bp fragment. (TIF)

S5 Fig *In silico* translation of FTH1P3 transcript. (TIF)
