# Supplementary figures and images for: FTH1P3, a Novel H-Ferritin Pseudogene Transcriptionally Active, Is Ubiquitously Expressed and Regulated during Cell Differentiation
Source: PLoS One. 2016 Mar 16;11(3):e0151359. doi: 10.1371/journal.pone.0151359 (PMC4794146; doi:10.1371/journal.pone.0151359)

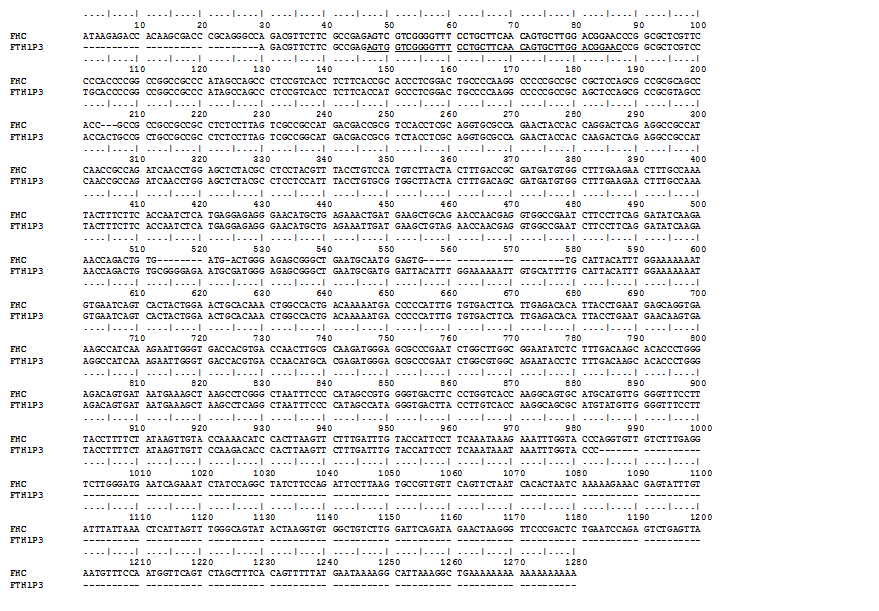

Supplement: S1 Fig — (TIF) [file pone.0151359.s001.tif]

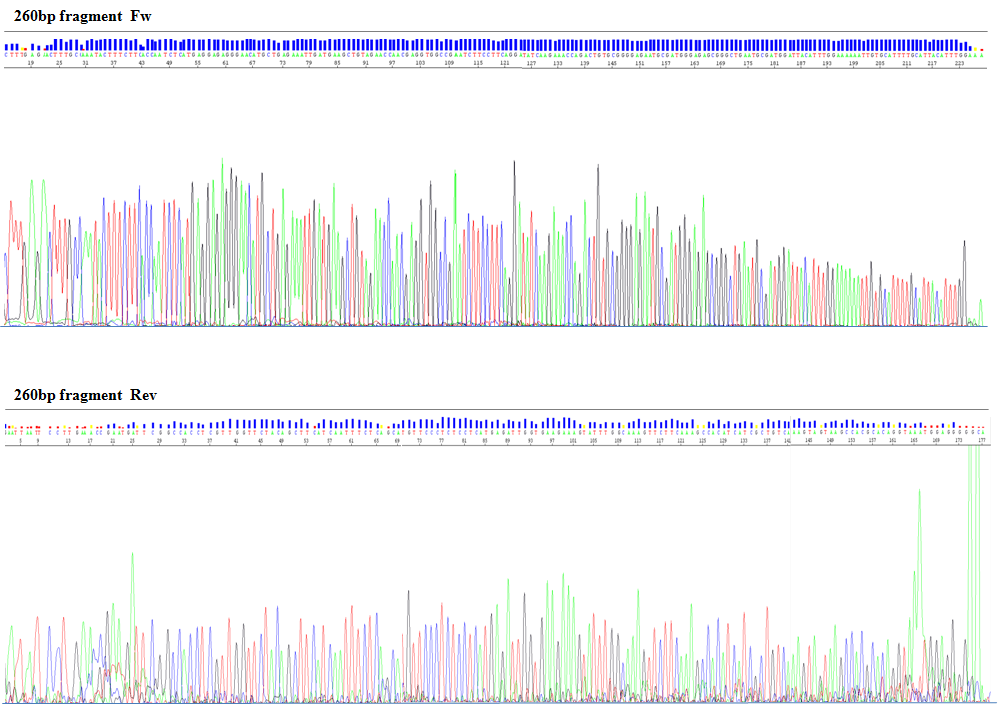

Supplement: S2 Fig — (TIF) [file pone.0151359.s002.tif]

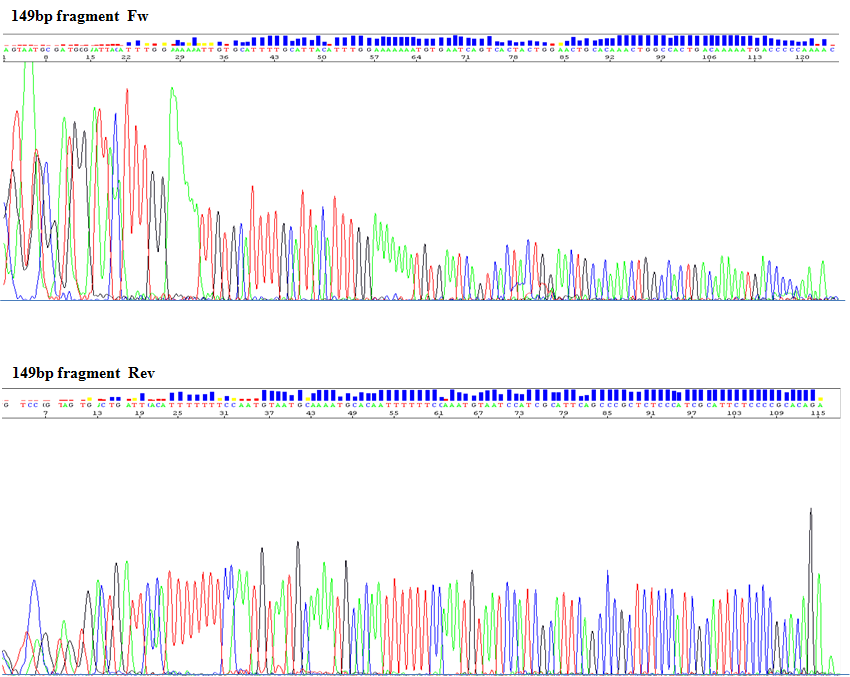

Supplement: S3 Fig — (TIF) [file pone.0151359.s003.tif]

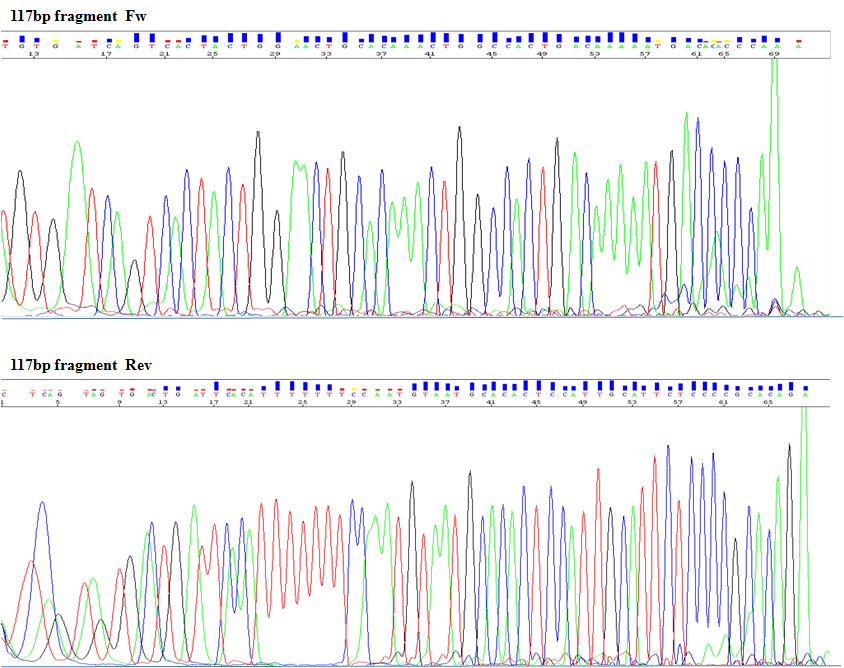

Supplement: S4 Fig — (TIF) [file pone.0151359.s004.tif]

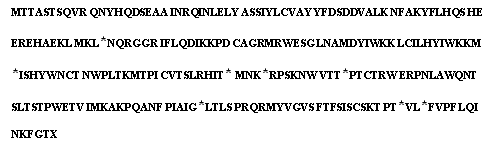

Supplement: S5 Fig — (TIF) [file pone.0151359.s005.tif]
